# Concussion is a temporary disability: rethinking mild traumatic brain injury in sports medicine

**DOI:** 10.3389/fneur.2024.1362702

**Published:** 2024-03-05

**Authors:** Zachary W. Bevilacqua

**Affiliations:** State University of New York at Brockport, Department of Kinesiology, Sport Studies and Physical Education, Brockport, NY, United States

**Keywords:** concussion, return to learn (RTL) services, college and university students, disability, 504 accommodation plan, patient care

## Introduction

Concussion, or mild traumatic brain injury, is not uniformly defined. Recent consensus describes concussion as a sport or exercise-related injury that perturbs neurotransmitter, metabolic, and axonal homeostasis (1). Other descriptions are more narrow in scope, specifying the signs and symptoms that must emerge following forceful trauma to the head (2). Iterative definitions of concussion are informed by impactful research, spurring new characteristics such as the utility of cerebral hemodynamics and functional magnetic resonance imaging in detecting abnormalities (1).

Generally, concussion is portrayed as an injury that imposes disabling sequela, however, the label of “disability” remains associated with the diagnoses of moderate and severe forms of traumatic brain injury (TBI) in published works (3, 4). Suspectedly, this is due to the brief, “mild” nature of concussion, with other disability diagnoses like severe TBI and attention deficit disorder representing lengthy or permanent impairments. While concussion was once thought to resolve in ≤ 2 and ≤ 4 weeks for adults and children, respectively (5), recent appraisal now indicates that a concise timeframe for recovery is difficult to determine (1). Regardless of its duration, concussion is a debilitating injury that is accompanied by a myriad of symptoms and functional impairments which substantially interrupt daily living. For instance, damage to vestibular and ocular connections can present obstacles when driving or engaging in school or work activities, whereas dysregulated cerebral blood flow will make basic activities difficult or uncomfortable. Deficiencies like these align with the Americans with Disabilities Act (ADA) definition of disability (6), and call into question why this or similar terms have not been woven into the mild TBI conversation. Accordingly, this article aims to begin the conversation by educating practitioners on why concussion is classified as a temporary disability, and more importantly, why interdisciplinary teams should consider using this language when managing concussion.

## What makes concussion a disability?

Disabilities are defined by the ADA as “(1) a physical or mental impairment that substantially limits one or more major life activities of an individual; (2) a record of such an impairment; or (3) being regarded as having such an impairment” (6). Under these standards, the Civil Rights Division of the United States Department of Justice saw fit to include traumatic brain injury in the 2016 amendments of the ADA. The statute reads:

“Traumatic brain injury substantially limits the condition or manner in which an individual's brain functions by impeding memory and causing headaches, confusion, or fatigue—each of which could constitute a substantial limitation on the major bodily function of brain function (6).”

The aim of the ADA is to put forth a definition of disability that is inclusive; therefore, as to not exclude those with transient traumatic brain injuries (concussion) from assistance, the statute amended the “*transitory and minor*” qualifier of its definition. It reads:

“The 6-month “transitory” part of the “transitory and minor” exception in paragraph (f)(2) of this section does not apply to the “actual disability” or “record of” prongs of the definition of “disability.” The effects of an impairment lasting or expected to last < 6 months can be substantially limiting within the meaning of this section for establishing an actual disability or a record of a disability (6).”

In short, a concussion that is diagnosed by a qualified healthcare provider and that limits a major life function (i.e., reading, sleeping, concentration, brain function, etc.) will satisfy both prongs of the definition for having a disability. These critical additions to a scoping piece of legislation are the first to recognize and address all forms of traumatic brain injury as a disability, possessing extensive implications in the workplace and on academic campuses. While the details will vary, ADA protection and concussion assistance is now applicable to professional athletes, student-athletes, and school-aged individuals, among others.

## Additions to concussion terminology

Since the late 1990's, language surrounding concussion has evolved, primarily shedding terms that incorrectly categorize the injury. For instance, the American Academy of Neurology (7) and Cantu (8) concussion scales classified concussion as mild (grade I), moderate (grade II), and severe (grade III). These descriptors were utilized for a number of years, but were departed from in support of the terms “simple” and “complex” to describe concussion (9). Again, novel insights would soon encourage the abandonment of this model, stating that “the terminology itself did not fully describe the entities (10).” Presently, concussion is uniformly viewed as a serious injury, with the length of recovery predicted primarily by the severity of post-injury symptom levels (11). Revisions to a working definition of concussion are anticipated, with an emphasis placed on the inclusion of evidence-based details. This article does not suggest that ADA language is the answer to effectively describing concussion in a way that previous models could not; rather, acknowledging concussion as a temporary disability will further outline the injury in an evidence-based manner, supporting the underlining goal of improving patient care. How this occurs is explained next using a college student as an example patient.

## Temporary disability support in concussion management

It is understandable that some may exhibit reluctance with pursuing disability support for patients with concussion, arguing that the term “temporary disability” inflates the effects of an injury that is expected to resolve in ≤ 4 weeks for most individuals. Why then is this language used when reporting on osseous injuries (12–15) despite the similar recovery time between fractures and concussion? Students with temporary orthopedic disabilities are provided with notetakers in class, access to elevators or restricted parking spaces, and leniency with commute time between classes. Equivalent accommodations are used by students with concussion (extended exam time, class attendance through Zoom videoconferencing, postponed deadlines, etc.), yet no association with disability status is mentioned. Accommodations in the university setting are issued by a central accessibility or disability office; however, if concussion is not realized as a disability, then healthcare providers, students, and others assisting in the student's recovery will be unaware that these institutional supports are applicable. Available evidence shows that 32% of sample university athletes (*n* = 1,974) received accommodations for their concussion (16), a meager value when considering the tuition, scholarship, degree progression, and course completion consequences that may result from concussion and missed instruction. These consequences are real, with 56% of sample college students reporting that a concussion would extremely limit their ability to complete an exam (17). The average student also agrees to $7,869 in public college tuition and fees each year, or $37,095 for private college (18), so the financial repercussions of falling behind academically are high. For further perspective, upwards of 38% of sample college students will not resume full academic participation within 2 weeks following concussion [females, 37.9% (19), 29.8% (16), 28.8% (20), 14.0% (20); males, 37.2% (19), 24.0% (16), 19.5% (20), 10.9% (20)]. At 1 month, 28.0% will still be on a return-to-learn path [females, 25.4% (19); males, 28.0% (19)], with 13.5% requiring ≥35 days to return-to-learn [females, 13.5% (16); males, 9.3% (16)]. These findings are worrisome, primarily when you consider that each week of a college semester accounts for roughly 7% of a course's content (e.g., 15-week semester, 3 h of instruction per week). Healthcare providers should strongly consider capitalizing on 504 accommodations for their college patients to ensure disability support is available during the anticipated weeks of recovery. Evidence indicates that college students would find this practice beneficial, with academic accommodations consistently identified as a positive addition to their concussion recovery (21–23). We should also keep in mind that 504 accommodations are not retroactive, meaning exams/assignments/etc. that are missed or negatively impacted by the student's concussion prior to 504 accommodations beginning will not be covered. This could have major implications for those who choose to wait-and-see how the student responds during the acute/subacute phase of recovery, or those who spend valuable time requesting informal academic adjustments from faculty members and are denied or are only partially supported. Researchers have previously summarized and validated that faculty are not always trusting of their students; thus without “official documentation” from a campus disability office, some faculty are reluctant, or even refuse, to provide accommodations for concussion (24, 25). Therefore, faculty see an “official” email from the disability office as a valuable piece to the academic management of concussion.

## Acquiring temporary disability support

A feasible approach to acquiring accommodations in the university setting has been previously outlined by Bevilacqua and McPherson (26), and utilizes a three-stage protocol to establish interdisciplinary collaboration and reproducible support for students. Specifically, the protocol encourages students suspected of head trauma be seen by a medical provider qualified to assess and diagnose concussion. From there, the patient can allow the provider to establish an open channel of communication (i.e., FERPA or HIPAA release form) with the disability office or similar entity on their college campus. Accessibility professionals are prepared to transform medical diagnoses into academic accommodations, can disseminate this information to faculty and staff, and possess an authoritative reputation on university campuses, making them ideal candidates as point personnel. These details, including guidance for patient follow-up are also discussed. The groundwork and processes for seamlessly ushering these patients toward disability accommodations are already in place and need only be utilized properly and efficiently to show success.

## What barriers exist?

Researchers have identified a number of factors that may influence care-seeking behavior following concussion (17, 27, 28), and by extension, students agreeing to disability support. This area of concussion inquiry is ongoing; however, we may possess some acumen in briefly discussing two specific barriers, namely student reluctance to alter academic participation following head trauma, and stigma surrounding receiving disability assistance.

College athletes identify their Athletic Trainer as a preferred outlet for disclosure (27); however, they also exhibit anxious tendencies toward missing classes and assignments that may result from disclosing their injury (29). Athletic Trainers report difficulty with college athletes adhering to cognitive rest, noting a greater acceptance of time away from physical activity (29). Authors acknowledge that college students may associate care-seeking with a restriction of their routine activities like school and exercise by the healthcare provider (17); yet this has not been adequately substantiated by the literature, nor does it align with longstanding concussion management practices to reintroduce normal activities no later than 48 h post-injury (1, 5). Should this be the case for some, however, dialog about temporary disability assistance could lessen patient anxiety and begin to change perceptions of concussion care from one of school restrictions to one of maintaining engagement with normal academic activities. Students should be told that 504 accommodations do not mandatorily reduce their academic participation, but instead allows the student to reintegrate at their own pace and with conscientious support from both faculty and staff. What's more, civil rights laws allow students to rest assured that accessibility orders will be upheld by faculty. Lastly, until terms like “disability assistance” and “temporary disability” become commonplace in concussion management, healthcare providers have the option to exercise caution or use more palatable language such as “academic accommodations” when speaking to their patients.

Students have also reported fear of stigmas associated with their disability. In fact, both systematic review (30) and subsequent large-scale data (*n* = 1,980) (31) indicate that stigma is an obvious concern for college students. Authors cite how students express fear of losing independence or suffering judgment from faculty. These concerns should be addressed as part of holistic concussion management, with providers conveying evidence that may ease the worries of these patients. For example, approaching faculty to discuss accessibility documents may seem daunting for students given the power dynamic between faculty and student; however, students could find relief in knowing that faculty view these types of accessibility notes as helpful when accommodations are needed (24, 25, 32). Additionally, while most accessibility requests span the length of a semester, support for concussion should be presented as an interim necessity, meaning that faculty will not be made to think that the student's performance is permanently altered. This should calm students who have anxieties of being viewed differently by faculty and staff, or who are nervous that academic opportunities may be withheld due to their injury (22). Lastly, providers should reiterate that the diagnosis of concussion is not shared with faculty unless the student allows, so students remain in control of their privacy.

## Conclusion

Per the ADA, concussion has been recognized as a temporary disability for the past several years, yet concussion literature has failed to address this newfound characteristic. This article fills this gap and confirms that concussion is viewed as something other than a transient sports injury. Providers are encouraged to familiarize themselves with disability terminology and the 504 resources available to patients suffering from concussion disability. These resources are particularly encouraged when discussing next steps with college students. While it was not reviewed here, other populations such as professional athletes and active individuals who experience concussion can benefit from ADA protection in the workplace. Disability terminology would be a progressive addition to future concussion talks and prompts management practices to consider the value-add of temporary disability assistance.

## Author contributions

ZB: Writing—original draft, Writing—review & editing.
